# From Crisis to Connectivity: Exploring the Role of Information and Communication Technologies in Medical Education During the COVID-19 Pandemic

**DOI:** 10.7759/cureus.60302

**Published:** 2024-05-14

**Authors:** Karina Ordaya-Gonzales, Juan Carlos Cortez Restuccia, Wilbert Juvenal Cossio Bolaños, Jose Arriola-Montenegro

**Affiliations:** 1 Department of Medicine, Universidad Cientifica del Sur, Lima, PER; 2 Department of Dentistry, Universidad Cientifica del Sur, Lima, PER; 3 Department of Stomatology, Escuela Profesional de Estomatologia, Universidad Privada San Juan Bautista, Lima, PER; 4 Department of Stomatology, Centro Medico Naval, Lima, PER; 5 Department of Medicine, University of Minnesota, Minneapolis, USA

**Keywords:** medical education curriculum, virtual setting, e-learning, covid-19, icts

## Abstract

Introduction: In a virtual setting at a private university in Lima, Peru, 277 medical students participated in a study in 2021 during the Coronavirus disease 2019 (COVID-19) pandemic.

Objective: The aim was to investigate how information and communication technologies (ICTs) were utilized as educational aids in their field.

Results: The findings showed a high level of satisfaction with ICT resources, especially among female students (54%). However, challenges were present: 64% faced technical issues during virtual classes, while 60% saw information availability and internet access as major advantages. Despite connectivity problems affecting 83% of students, 55% believed ICTs supported collaborative learning. Interestingly, while 64% found ICT use distracting, 52% found it easy to use.

Conclusion: ICTs played a significant role in medical education, introducing new methods and tools despite obstacles and providing a dynamic and adaptable e-learning environment.

## Introduction

During the context of the Coronavirus disease 2019 (COVID-19) pandemic, education worldwide has been compelled to innovate and adapt, prioritizing virtual tools to prevent learning delays and achieve educational goals set by educational institutions [1]. For example, the higher education sector has implemented virtual classes and embraced e-learning tools, with virtual platforms, online simulations, and simple questionnaires being the most commonly used [2]. This has led to a continuous reliance on information and communication technologies (ICTs) by both students and teaching staff [3].

For students, the availability of information and collaborative learning have been significant mediators in this new virtual world, where students consider ICTs as tools for interaction among multiple participants within the teaching and learning process [4]. In fact, it is argued that during this context, students have developed a positive perspective on the use of digital tools, concluding that they make a significant contribution to facilitating communication with teachers and resolving doubts during class, resulting in a quick and positive adaptation to the new reality [5]. Therefore, ICTs are considered potential tools for students' skill acquisition and are currently seen as an important educational resource [6]. Authors such as Prieto et al. recommend the use of free digital tools in the classroom, such as Mentimeter, Kahoot, and Socrative, as they are accessible and only require a Wi-Fi connection. Furthermore, these digital tools promote autonomous and collaborative learning, generate high motivation, can be used individually or in small groups, and allow for real-time responses, enabling teachers to provide immediate feedback to students [7].

According to some authors, medical students have achieved significant learning outcomes through the use of ICTs, characterized by Zoom sessions that include supervised work sessions [8]. Similarly, other authors have observed benefits with the use of ICTs, indicating that their purely academic use brings great benefits to students in the field of Human Medicine, improving teamwork, developing technological skills, allowing for creative presentations, and generating a greater interest in virtual forums, motivating them to create shared virtual spaces where they can exchange information without temporal barriers [9]. However, other studies have identified a negative perception among students regarding virtual learning in the context of the COVID-19 pandemic, which could be attributed to external factors such as environmental distractions or added noises during distance online classes, student-related factors such as difficulties in developing soft skills in practical courses that involve social interaction, and even knowledge and access to ICTs [10-12]. 

The present study sought to describe the use of ICTs as an educational tool in higher education, specifically in the field of Human Medicine, with the aim of providing information and data found during the COVID-19 pandemic. Thus, it aims to serve as a basis for future research and motivate new strategies to improve the implementation of ICTs in virtual education.

## Materials and methods

Study design

The study conducted was quantitative, observational, descriptive, and cross-sectional. The researchers did not intervene in the study population; they solely observed the prioritized phenomena and described the results according to the study objectives, with data collection carried out at a single point in time.

Population

The population consisted of 277 university students aged 18 to 25, enrolled in the II-VII cycles of study (each academic year consisting of two cycles), who attended virtual classes during 2021 at a private university in Lima, Peru. All 277 students were included in the study.

Data collection

A structured questionnaire was developed, consisting of closed and pre-coded questions. The items or phrases used to measure variables or constructs were adapted from a previous article [13,23]. The questionnaire comprised 28 questions divided into four dimensions: general data, study filter data, category habits, and control data. The questionnaire was validated through a pilot test, with reliability assessed using Cronbach's internal consistency coefficient with α > 0.7%. This was done to ensure that all objectives of the study were covered, verify logical sequence, and ensure proper understanding of the questions.

Data analysis

The survey data collected from Google Forms was exported to Microsoft Excel 2017, where variables were coded to facilitate analysis. Absolute and relative frequency measures were used to describe categorical variables. Finally, tables and graphs were created based on the obtained information, with a focus on the most significant findings related to the use of ICTs as an educational tool.

Inclusion and exclusion criteria

(1) Participants must be over 18 years old. (2) Participants must be enrolled in the Human Medicine Career at Universidad Científica del Sur in Lima, Peru. (3) Participants must have been taking virtual classes during the year 2021.

## Results

In Table 1, the sociodemographic characteristics reveal that 97% of the population were single. Female medical students predominated at 54%, while male students accounted for 46%. The predominant age range was between 18 and 21, comprising 58% of the total. Furthermore, the majority were enrolled in the fourth cycle of the Human Medicine program. In terms of access to technological devices, laptops were the most commonly used, with a consistent daily frequency and stable internet connection.

**Table 1:** Demographic aspects Own elaboration based on the investigated data. The items or phrases used to measure variables or constructs were adapted from a previous article [13,23]. Absolute count: number of students. Percentage (%): representation of the proportion of students in relation to the total sample.

Demographic aspects		
Variable	Absolute count	Percentage (%)
Gender		
Male	128	46%
Female	149	54%
Total	277	100%
Age		
18 to 21 years	162	58%
22 to 24 years	73	26%
25 to 27 years	35	13%
28 to 30 years	7	3%
Total	277	100%
Study cycle		
Second - II	3	1%
Third - III	8	3%
Fourth - IV	122	44%
Fifth - V	93	34%
Sixth - VI	37	13%
Seventh - VII	14	5%
Total	277	100%
Multimedia device		
Laptop	210	76%
Tablet	172	62%
Desktop computer	157	57%
Mobile phone	129	47%
Others	8	3%
Total	277	244%
Frequency of platform use		
Every other day	34	12%
Every day	232	84%
Once a week	11	4%
Total	277	100%
Internet connection		
No	3	1%
Yes	274	99%
Total	277	100%
Marital status		
Married	7	3%
Single	270	97%
Total	277	100%

According to the results from Table 2, nearly nine out of 10 respondents agree that the use of ICTs has been easy in a virtual class, with a higher percentage among students aged 18 to 21 years old. Likewise, almost nine out of 10 respondents concur that ICTs have been easy to learn and simplify task completion. Additionally, almost eight out of 10 agree that they accelerate work in class. More than two-thirds of respondents accept that using ICTs facilitates social interaction, while nearly six out of 10 agree that they increase student productivity. However, less than half perceive difficulty in performing group work during virtual classes.

**Table 2:** Top two box of the use of ICTs Table 2 is a "top two box," which involves summing the percentage of responses in these two categories to obtain an overall measure of satisfaction or approval. This metric is used to simplify analysis and provide a clearer measure of positive perception. Percentage (%): representation of the proportion of students in relation to the total sample. ICTs, information and communication technologies

Use of ICTs								
	Gender		Age			Cycle		Total general
	Male	Female	18 to 21	22 to 24	25 to 30	II-III-IV	V-VI-VII	
Considers that the use of ICTs has been easy to use in a virtual class	82%	91%	92%	85%	71%	92%	83%	87%
Considers that the use of ICTs has been easy to learn	84%	88%	90%	85%	74%	88%	84%	86%
Using ICTs simplifies performing a task or assignment	82%	88%	91%	82%	69%	89%	82%	85%
Using ICTs speeds up work in class	77%	81%	85%	77%	62%	83%	76%	79%
Finds it easier to socially interact in a virtual class compared to an in-person one	68%	74%	80%	68%	45%	77%	67%	71%
Considers that the use of ICTs increases student productivity	56%	61%	59%	63%	52%	68%	51%	59%
Perceives difficulty in performing group work during virtual classes	19%	24%	12%	33%	38%	14%	28%	22%

Table 3 shows the following results: 64% of respondents experienced technical problems, 56% of students had insufficient internet signal, and 43% faced difficulty in receiving feedback in an online class compared to a traditional class. Additionally, 42% believe there is difficulty in motivating students in the online environment, and 35% perceive that the level of interactions in online courses is lower than in traditional ones. Moreover, 23% commented on a scarcity of didactic material, and 21% consider there is a heavy workload in online courses.

**Table 3:** Barriers and challenges of using ICTs Absolute count: number of students; percentage (%): representation of the proportion of students in relation to the total sample. ICTs, information and communication technologies

Barriers and challenges of using ICTs		
	Absolute count	Percentages, %
Technical problems	178	64%
Insufficient internet signal	156	56%
Difficulty in receiving feedback in an online class compared to a traditional class	119	43%
Difficulty in dividing into subgroups for group work tasks	116	42%
Difficulty in motivating students in the online environment	115	42%
The level of interactions in online courses is lower than in traditional ones	98	35%
Long time to complete tasks online	88	32%
Perceived scarcity of didactic material in the class	65	23%
The heavy workload of online courses	57	21%

Table 4 shows that 60% of respondents consider the availability of information as an advantage, 55% believe there is greater collaborative learning, and 52% consider there is greater learning of the discipline. Additionally, 49% believe there is increased interaction with the teacher and 49% with peers, while 23% consider there is greater communication between the teacher and students. On the other hand, regarding disadvantages, the table shows the following results: 83% experienced connection problems, 64% of respondents felt that they had access to many distractions, and 16% consider there is less teacher-student communication. 

**Table 4:** Advantages and disadvantages offered by the ICTs Absolute count: number of students. Percentage (%): representation of the proportion of students in relation to the total sample. ICTs, information and communication technologies

Advantages and disadvantages offered by the ICTs		
	Absolute count	Percentages, %
Advantages		
Availability of information	166	60%
Collaborative learning	151	55%
Increased interaction with the teacher	145	52%
Greater learning of the discipline	137	49%
Increased interaction with peers	136	49%
Greater communication (teacher-student)	65	23%
Disadvantages		
Connection failure	230	83%
Access to many distractions	178	64%
Less teacher-student communication	44	16%
Lack of interaction with peers	43	16%
Less learning	37	13%

Table 5 shows that 52% consider their proficiency in ICTs as excellent, while 50% agree that handling technological tools has been very easy. Additionally, 63% of respondents believe that teachers have a regular level of proficiency in technological tools, and 58% reported being completely satisfied with the use of virtual platforms used in the university. Likewise, 38% of respondents felt that the teacher establishes conducive learning environments using ICTs very frequently, and 45% use computers and other information technologies very frequently when giving presentations in class.

**Table 5:** Factors indicating the acceptance of ICTs Absolute count: number of students. Percentage (%): representation of the proportion of students in relation to the total sample. ICTs, information and communication technologies

Factors indicating the acceptance of ICTs		
	Absolute count	Percentages (%)
Proficiency in ICTs		
Excellent	145	52%
Very good	81	29%
Regular	51	18%
Difficulty in using technological tools		
Very easy	139	50%
Easy	79	29%
Neutral	50	18%
Difficult	6	2%
Very difficult	3	1%
Teaching staff proficiency in technological tools		
Regular	175	63%
Excellent	97	35%
Low	5	2%
Satisfaction with the use of virtual platforms in the university		
Completely satisfied	161	58%
Quite satisfied	84	30%
Indifferent	26	9%
Quite dissatisfied	4	1%
Completely dissatisfied	2	1%
Establishment of conducive environments by the teacher in using ICTs		
Very frequent	106	38%
Quite frequent	78	28%
Frequently	73	26%
Infrequently	17	6%
Never	3	1%
Use of ICTs while giving presentations in class		
Very frequent	124	45%
Quite frequent	84	30%
Frequently	61	22%
Infrequently	6	2%
Never	2	1%

## Discussion

The population profile outlined in Table 1 highlights the gender distribution in ICT usage, with 46% being male and 54% female. This aligns with Aguilar et al.'s research, indicating that women demonstrate superior performance and adaptation in utilizing ICT compared to men [13]. Additionally, the majority of the participants belonged to the age group of 18 to 21 years, accounting for 58% of the population. However, this age gap can be addressed by educational institutions, as it has been shown that with training strategies, the adaptation to the use of ICT can be feasible even at older ages, as evidenced by the study conducted by Campos et al., who stated that age is not a barrier to the use of ICT [14,15]. Regarding the study cycles, there was a higher participation of students in the fourth and fifth cycles, accounting for 44% and 34%, respectively, which has been reported in previous studies, as this age group tends to have continuous use of ICT for social communication, making it easier for them to adapt its use in other areas [16]. It was also found that younger people are regularly exposed to technology, using it as a tool for information and knowledge [13].

Regarding the use of connectivity devices, the most commonly used connection method in the present study was the use of laptops, with a daily usage frequency of 76%, followed by tablets. While it is known that according to the National Institute of Informatics, cell phones are the most commonly used technology in Peru, it is expected that this may not be the case in the educational context due to the smaller screen size, lower audio quality, and limited availability of functional applications, making it not the first choice for virtual education [17]. However, in certain populations where access to tablets or laptops may not be possible due to high costs, cell phones can be a feasible option utilized for virtual education, as mentioned by Walsh et al. [18]. Finally, it is important to recognize that the use of ICT is closely related to student accessibility, both for acquisition and use [19]. This is consistent with the findings of the research conducted by Campos et al., who identified that the lack of technological equipment and limited training led to lower satisfaction, which was also observed in the population of this study, considering the particular characteristics of the population, as they are young students with a greater ease of using ICT due to continuous use in other areas of their daily lives [20].

According to the results of Table 2, which corresponds to the objectives of perceptions and experiences regarding the use of ICT, it was observed that approximately nine out of 10 interviewees consider them easy to use in a virtual class. This is more common among women, accounting for 91%, with ages between 18 and 21 accounting for 92%, and in the academic cycles from II to IV accounting for 92%. Therefore, the population considered mastery of ICT to be excellent, making work or tasks easier to accomplish. All of the above is related to the findings of a research study conducted by a private university in Lima, which shows that ICT has been easy to use and has generated changes in the digital ecosystem, simplifying the processes of learning and teaching in virtual environments [21].

Similarly, nine out of 10 interviewees reported that the use of ICT has been easy to learn, with a higher frequency among women accounting for 88%, and ages between 18 and 21 accounting for 90%, confirming the research conducted by Almenara et al., who demonstrate that ICT promotes the development of teaching and learning processes, allowing students to have different alternatives to achieve learning objectives more easily [22]. Therefore, in this study, they are considered virtual tools that provide many opportunities for their use and learning.

In addition, it was found that approximately six out of 10 interviewees considered that the use of ICT increases student productivity. This perception was more frequent among women, accounting for 61%, with ages between 22 and 24 accounting for 63%, and in academic cycles from II to IV accounting for 68%. This supports the research by Zalat et al., where it was found that 77.1% of students increased their productivity with the use of ICT during virtual classes [23]. Similarly, it was identified that only 22% perceived difficulty in using ICT. This difficulty was mainly reported by women, accounting for 24%, with ages between 25 and 30 accounting for 38%, and in academic cycles from V to VI accounting for 28%. This supports the research by Aguilar et al., who stated in their study that 25% of participants perceived difficulty in using ICT. Therefore, it was evident that ICT was shown as an easily usable strategy in our study, taking into account that it is not necessarily observed in all cases of students in virtual education, especially in developing countries like Peru, where there is a heterogeneity of knowledge in the use of ICT that is related to socioeconomic status, region, and rurality [24].

Table 3 corresponds to the barriers and challenges encountered in the use of ICT, showing that 64% of the respondents experienced technical problems, 56% of students had insufficient internet signal, and 43% faced difficulty in receiving feedback in an online class compared to a traditional class. This is supported by the research conducted by Martin et al., which states that a barrier and challenge in the use of ICT are problems with internet signal, which in some cases makes its use impossible [25]. Similarly, it is mentioned in the study conducted by Zalat et al., which shows that 32.1% of students experienced technical problems, 40.2% had issues with the internet signal, and 42% had problems with receiving feedback from teachers in online classes [23].

Furthermore, 42% of students consider that there is difficulty in motivating students in the online environment, and 35% believe that the level of interactions in online courses is lower than in traditional ones. Additionally, 23% comment on the scarcity of didactic materials, and 21% consider that there is a heavy workload in online courses. These results are consistent with the findings of the research by Zalat et al., who reported that 16.2% had low motivation in virtual development, 24.3% perceived little interaction in its use, and 19.9% found insufficient didactic material in the development of online classes [23]. These considerations should be evaluated by educational institutions when implementing virtual education strategies in order not to disadvantage students who face any of these barriers.

Finally, this study showed one of the barriers to be the difficulty in motivating students in a virtual environment. However, this result does not align with what was mentioned in the study by Paulin et al., where it is stated that there are platforms within ICT that could be used due to their easy accessibility and usability, which motivates students' interest and enhances their work or study on an assigned topic, improving collaborative learning [26]. This discrepancy in the results from previous studies may be due to the abrupt implementation of virtual education in Peru due to the COVID-19 pandemic, which resulted in a lack of planning to address virtuality in education, unlike other contexts where ICT was commonly used in education, and virtuality was a usual learning strategy [19].

## Conclusions

While the higher education sector rapidly implemented online classes and e-learning tools leading to significant advancements in ICT, it can be concluded that there has been effective utilization of ICT in educational processes, underscoring their importance. Both students and educators share responsibility for maximizing the benefits of these resources. However, it is imperative for educational institutions to refine ICT usage planning in teaching and learning, including regular assessment of student satisfaction.

This study concludes that ICT use has made a positive contribution to education by introducing new methods and technological tools. It was observed that laptops and tablets were the most commonly utilized devices, particularly among 18- to 21-year-old populations, highlighting prominent sociodemographic factors. Technical issues and internet connectivity were identified as major barriers and challenges to ICT use. Additionally, the availability of information emerged as a significant advantage that positively impacted students.

Most students reported a favorable perception of ICT implementation within the medical curriculum, demonstrating proficiency and ease of use. However, significant barriers such as the presence of distractions need to be addressed to enhance student satisfaction and accessibility to ICT in the university setting.
